# Impact of spontaneous abortion history and induced abortion history on perinatal outcomes of singleton pregnancies

**DOI:** 10.1186/s12889-023-17264-5

**Published:** 2023-11-29

**Authors:** Hanxiang Sun, Jing Mao, Xiujuan Su, Qiaoling Du

**Affiliations:** 1 Department of Obstetrics, Shanghai Key Laboratory of Maternal Fetal Medicine, Shanghai Institute of Maternal-Fetal Medicine and Gynecologic Onclogy, Shanghai First Maternity and Infant Hospital, School of Medicine, Tongji University, Shanghai, 200092 China; 2 Clinical Research Center, Shanghai Key Laboratory of Maternal Fetal Medicine, Shanghai Institute of Maternal-Fetal Medicine and Gynecologic Onclogy, Shanghai First Maternity and Infant Hospital, School of Medicine, Tongji University, Shanghai, 200092 China

**Keywords:** Spontaneous abortion, Induced abortion, Singleton pregnancy, Perinatal outcome

## Abstract

**Background:**

At present, there are several studies on abortion history and perinatal outcomes, but there is no unified conclusion whether the history of abortion and different types of abortion are related to perinatal complications of subsequent pregnancy. We aim to study the impact of different types of abortion history on perinatal outcomes of singleton pregnancies.

**Methods:**

This was a retrospective study from a maternity and infant hospital in Shanghai, China from 2016 to 2020. Pregnant women who gave birth to live singleton infant were included (*n* = 75,773). We classified abortion into spontaneous abortion (SAB) and induced abortion (IA). We compared the perinatal outcomes of singleton pregnancies with different abortion histories and used Logistic regression analysis to evaluate the associations between pre-pregnancy abortion history with perinatal outcomes.

**Results:**

We observed that pregnant women with a history of abortion were more likely to have a premature delivery (0.99% VS 0.45%), gestational diabetes mellitus (GDM) (13.40% VS 10.29%), placenta abnormality (8.16% VS 5.06%), placenta previa (5.65% VS 3.75%), placenta accreta (0.18% VS 0.04%), and placenta adhesion (2.79% VS 1.03%) than those who obtained singleton pregnancies without a history of abortion. When confounding factors were adjusted, differences in placenta abnormality still existed (excluding placenta abruption). The odds ratios and 95% confidence interval of placenta previa, placenta accreta, and placenta adhesion in pregnant women with only SAB history, only IA history, and both abortion history were 1.294(1.174–1.427), 1.272(1.159–1.396), and 1.390(1.188–1.625), 2.688(1.344–5.374), 2.549(1.268–5.125), and 5.041(2.232–11.386), 2.170(1.872–2.515), 2.028(1.738–2.366), and 3.580(2.917–4.395), respectively.

**Conclusions:**

Our research showed that pregnant women who have a history of abortion before pregnancy were more likely to have premature birth, GDM, placenta previa, placenta accreta, and placenta adhesion. After adjusting for confounding factors, we found that the history of SAB, IA, and both SAB and IA history were related to the increased risk of placenta previa, placenta accreta, and placenta adhesion.

## Introduction

Abortion services have made progress in recent decades. In particular, the development of medical abortion can facilitate an early abortion for women. But complications caused by abortion are still an important cause of maternal mortality worldwide [1–3]. Research data in the United States suggested that abortion affected 1/3 of women each year. And by the age of 45, one in four American women has had an abortion [4, 5]. Therefore, it is necessary to continue the research related to abortion. According to the classification of abortion, it can be divided into spontaneous abortion and induced abortion. There is no unified conclusion on whether the history of abortion and different types of abortion are related to perinatal complications of the subsequent pregnancy.

At present, there are some studies on abortion and perinatal outcomes worldwide, but the conclusions are different. A study in the United States believed that there was a strong connection between the first abortion and the possibility of a second abortion in subsequent pregnancy [6]. According to a study from Britain, pregnant women with a history of abortion were more likely to have preeclampsia, premature delivery, and low birth weight in a subsequent pregnancy than those without a history of abortion [7]. Liran Hiersch et al. found that pregnant women with a history of miscarriage were more likely to develop GDM in subsequent pregnancies. Multivariate analysis showed that the history of abortion would lead to a higher cesarean section rate and placental retention rate [8]. Some studies have also explored the association between IA and pregnancy outcomes. A Finnish study explored the association of abortion history with obstetric pregnancy outcomes. The study found that premature delivery and low birth weight were more common among women who had abortions, but after Logistic regression analysis, they found no correlation between them, so the study considered that abortion was not an independent risk factor for obstetric adverse outcomes [9]. However, Alison Lowit and others from Britain believed that the history of IA was related to the increased risk of placenta previa, placental abruption, and low birth weight [10]. In addition, there were some studies on SAB history and pregnancy outcomes, especially on recurrent abortion, but the relevant conclusions were not unified [11, 12].

In view of the fact that there is no unified conclusion on the study of whether abortion history and different types of abortion history are related to perinatal complications of subsequent pregnancy. Therefore, this study was based on the large delivery volume of the class A tertiary obstetrics and gynecology hospital in Shanghai, China, and explored the association between the obstetrical outcomes of subsequent pregnancy with only SAB history, only IA history, and both history of SAB and IA.

## Materials and methods

### Study population

**Fig. 1:**
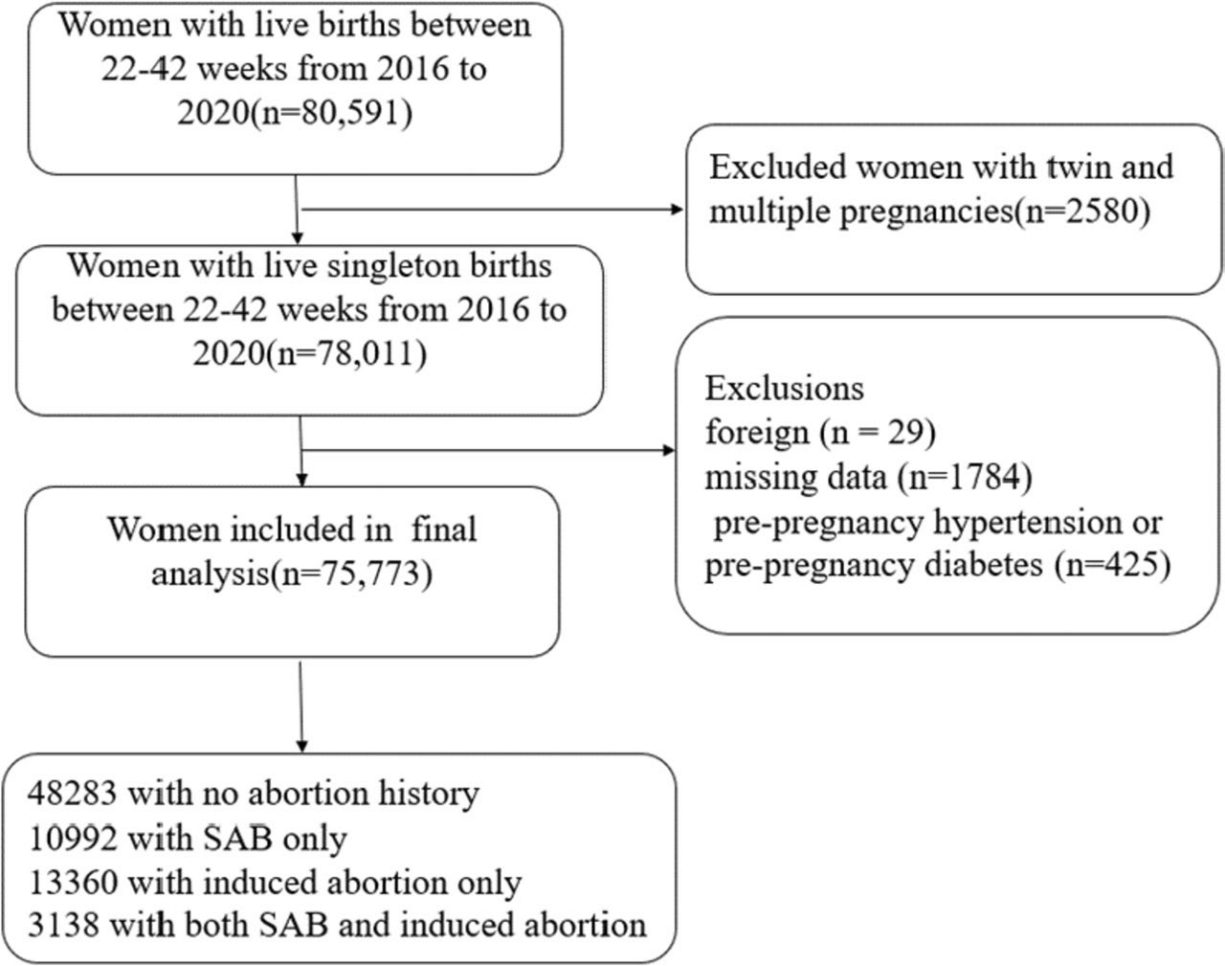
Flow chart of the participant recruitment process. (Abbreviation: SAB, spontaneous abortion)

This was a retrospective study at a class A tertiary obstetrics and gynecology hospital in Shanghai, including women who obtained singleton live pregnancies in this hospital from 2016 to 2020 (*n* = 75,773). Women with twin and multiple pregnancies (*n* = 2,580), foreigners (*n* = 29), missing data (*n* = 1,784), pre-pregnancy hypertension or pre-pregnancy diabetes (*n* = 425) were excluded from the study. At last, a total of 75,773 women with live singleton births were included for analysis. Among them, 48,283had no history of abortion, 10,992 had only a history of SAB, 13,360had only a history of IA, and 3,138 had both a history of SAB and a history of IA (Fig. 1).

### Exposure

The primary explanatory variable was the history of abortion, and it was classified as only SAB history, only IA history, both SAB history and IA history, and no abortion history.

### Outcomes

We were interested in perinatal complications, including placenta abnormality, premature delivery, GDM, pregnancy hypertension disorder, intrahepatic cholestasis of pregnancy, and oligohydramnios. Placenta abnormality included placenta previa, placenta abruption, placental accreta, and placental adhesion. Placenta previa means that the lower edge of the placenta reaches or covers the cervix, which is lower than the fetal presentation. Placenta abruption means that the placenta is partially or completely stripped from the uterine wall before the fetus is delivered. Placenta accreta means that placental villi penetrate the myometrium of the uterine wall. Placental adhesion means that the placenta adheres to the uterine wall in whole or in part and cannot be peeled off by itself. Pregnancy hypertension disorder included PIH and preeclampsia.

### Statistical analysis

The basic characteristics of the study population were statistically analyzed by descriptive analysis. Count (%) was used for categorical variables. The cross table was used to test whether women with an abortion history were related to perinatal complications compared with women without an abortion history. *P* < 0.05 is considered statistically significant. Logistic regression analysis was used to estimate the associations between different classifications of abortions and adverse perinatal outcomes. Possible confounding factors included maternal age, pre-pregnancy BMI (< 18.5 kg/m^2^, 18.5 kg/m^2^- < 25 kg/m^2^, ≥ 25 kg/m^2^) [13], mode of delivery (vaginal delivery or cesarean section) and parity (primipara or multipara). The classification of BMI was based on the standard of WHO [13].

All analyses were processed by the SPSS26.0 software package (SPSS Inc, Chicago, IL, USA).

## Results

Among the 75,773 included pregnant women, 48,283(63.72%) had no history of abortion, 10,992(14.51%) had only a history of SAB, 13,360(17.63%) had only a history of IA, and 3,138 (4.14%) had both history of SAB and history of IA. Compared with pregnant women without an abortion history, pregnant women with an abortion history were more likely to be old (age ≥ 35 years old) (23.02% VS 9.67%), and the probability of BMI ≥ 25 kg/m^2^ before pregnancy is greater (10.27% VS 7.94%). Moreover, pregnant women with a history of abortion had a higher probability of cesarean section (38.62% VS 29.20%), and the probability of multipara was higher (37.01% VS 17.55%). In addition, women with a history of abortion had a higher risk of placenta abnormality (8.16% VS 5.06%) (including placenta previa (5.65% VS 3.75%), placenta accreta (0.18% VS 0.04%), and placenta adhesion (2.79% VS 1.03%)), premature delivery (0.99% VS 0.45%), and GDM (13.40% VS 10.29%). However, there was no statistical difference in the incidence of placenta abruption (0.42% VS 0.51%), pregnancy hypertension disorder (4.53% VS 4.50%), intrahepatic cholestasis of pregnancy (1.12% VS 1.18%) and oligohydramnios (1.25% VS 1.22%). The detailed information of the research population are shown in Table 1.

**Table 1:** Basic characteristics and adverse outcomes of the study population by the history of abortion or not

Age, y, n (%)	History of abortion (*n* = 27,490)	No abortion history (*n* = 48,283)	*P* value
≤ 24	885 (3.22)	2,195 (4.55)	0.000
25–29	8,411 (30.60)	22,284 (46.15)	
30–34	11,866 (43.16)	19,137 (39.64)	
≥ 35	6,328 (23.02)	4,667 (9.67)	
Pre-pregnancy BMI, n (%)			
< 18.5 kg/m^2^	3,592 (13.08)	7,768 (16.09)	0.000
18.5 kg/m^2^- < 25 kg/m^2^	21,074 (76.66)	36,681 (75.97)	
≥ 25 kg/m^2^	2,824 (10.27)	3,834 (7.94)	
Parity, n (%)			
Nulliparous	17,316 (62.99)	39,811 (82.45)	0.000
Multiparous	10,174 (37.01)	8,472 (17.55)	
Mode of delivery, n (%)			
Vaginal delivery	16,873 (61.38)	34,185 (70.80)	0.000
Cesarean section	10,617 (38.62)	14,098 (29.20)	
Placenta abnormality, n (%)	2,243 (8.16)	2,442 (5.06)	0.000
Placenta previa, n (%)	1,552 (5.65)	1,810 (3.75)	0.000
Placenta abruption, n (%)	116 (0.42)	244 (0.51)	0.109
Placental accreta, n (%)	50 (0.18)	17 (0.04)	0.000
Placental adhesion, n (%)	767 (2.79)	497 (1.03)	0.000
Premature birth, n (%)	271 (0.99)	217 (0.45)	0.000
Gestational diabetes mellitus, n (%)	3,684 (13.40)	4,966 (10.29)	0.000
Pregnancy hypertension disorder, n (%)	1,245 (4.53)	2,172 (4.50)	0.846
PIH, n (%)	626 (2.28)	1,006 (2.08)	0.077
Preeclampsia, n (%)	619 (2.25)	1,166 (2.42)	0.154
Intrahepatic cholestasis of pregnancy, n (%)	309 (1.12)	572 (1.18)	0.454
Oligohydramnios, n (%)	344 (1.25)	591 (1.22)	0.743

We studied the association between different types of abortion and perinatal complications of singleton pregnancies and found that compared with pregnant women without abortion history, those with abortion history (including SAB history or IA history) were more likely to have premature delivery, GDM, pregnancy hypertension disorder, placenta previa, placenta accreta, and placenta adhesion, and there were statistical differences (*P* < 0.05). Compared with pregnant women without abortion history, women with a history of SAB were more likely to have premature delivery (0.76% VS 0.45%), GDM (15.00% VS 10.29%), pregnancy hypertension disorder (5.41% VS 4.50%), placenta abnormality (8.04% VS 5.06%), placenta previa (5.43% VS 3.75%), placental accreta (0.15% VS 0.04%) and placental adhesion (2.88% VS 1.03%), and there was a statistical difference (*P* < 0.05). Women with a history of IA were more likely to have premature delivery (1.04% VS 0.45%), GDM (11.75% VS 10.29%), pregnancy hypertension disorder (3.81% VS 4.50%), placenta abnormality (7.69% VS 5.06%), placenta previa (5.63% VS 3.75%), placental accreta (0.16% VS 0.04%) and placental adhesion (2.30% VS 1.03%). The difference was also statistically significant (*P* < 0.05). However, we did not observe the statistical difference between women with a history of both SAB and IA and women without a history of abortion in pregnancy hypertension disorder. The details were shown in Table 2.

**Table 2:** Basic characteristics and adverse outcomes of the study population by different classifications of abortions

			History of abortion				No abortion history (*n* = 48,283)
SAB only (*n* = 10,992)		*P* value^a^	Induced abortion only (*n* = 13,360)	*P* value^a^	Both SAB and induced abortion (*n* = 3,138)	*P* value^a^	
Age, y, n (%)							
≤ 24	231 (2.10)	0.000	588 (4.40)	0.000	66 (2.10)	0.000	2,195 (4.55)
25–29	3,286 (29.89)		4,427 (33.14)		698 (22.24)		22,284 (46.15)
30–34	5,240 (47.68)		5,256 (39.34)		1,370 (43.66)		19,137 (39.64)
≥ 35	2,235 (20.33)		3,089 (23.12)		1,004 (31.99)		4,667 (9.67)
Pre-pregnancy BMI, n (%)							
< 18.5 kg/m^2^	1,293 (11.76)	0.000	1,960 (14.67)	0.000	339 (10.80)	0.000	7,768 (16.09)
18.5 kg/m^2^- < 25 kg/m^2^	8,472 (77.07)		10,173 (76.14)		2,429 (77.41)		36,681 (75.97)
≥ 25 kg/m^2^	1,227 (11.16)		1,227 (9.18)		370 (11.79)		3,834 (7.94)
Parity, n (%)							
Nulliparous	8,551 (77.79)	0.000	7,124 (53.32)	0.000	1,641 (52.29)	0.000	39,811 (82.45)
Multiparous	2,441 (22.21)		6,236 (46.68)		1,497 (47.71)		8,472 (17.55)
Mode of delivery, n (%)							
Vaginal delivery	6,737 (61.29)	0.000	8,374 (62.68)	0.000	1,762 (56.15)	0.000	34,185 (70.80)
Cesarean section	4,255 (38.71)		4,986 (37.32)		1,376 (43.85)		14,098 (29.20)
Placenta abnormality, n (%)	884 (8.04)	0.000	1028 (7.69)	0.000	331 (10.55)	0.000	2,442 (5.06)
Placenta previa, n (%)	597 (5.43)	0.000	752 (5.63)	0.000	203 (6.47)	0.000	1,810 (3.75)
Placenta abruption, n (%)	46 (0.42)	0.239	54 (0.40)	0.136	16 (0.51)	0.972	244 (0.51)
Placental accreta, n (%)	17 (0.15)	0.000	21 (0.16)	0.000	12 (0.38)	0.000	17 (0.04)
Placental adhesion, n (%)	316 (2.88)	0.000	307 (2.30)	0.000	144 (4.59)	0.000	497 (1.03)
Premature birth, n (%)	84 (0.76)	0.000	139 (1.04)	0.000	48 (1.53)	0.000	217 (0.45)
Gestational diabetes mellitus, n (%)	1649 (15.00)	0.000	1570 (11.75)	0.000	465 (14.82)	0.000	4,966 (10.29)
Pregnancy hypertension disorder, n (%)	595 (5.41)	0.000	509 (3.81)	0.001	141 (4.49)	0.989	2,172 (4.50)
PIH, n (%)	308 (2.80)	0.000	245 (1.83)	0.068	73 (2.33)	0.362	1,006 (2.08)
Preeclampsia, n (%)	287 (2.61)	0.230	264 (1.98)	0.003	68 (2.16)	0.379	1,166 (2.42)
Intrahepatic cholestasis of pregnancy, n (%)	118 (1.07)	0.327	144 (1.08)	0.308	47 (1.50)	0.119	572 (1.18)
Oligohydramnios, n (%)	143 (1.30)	0.510	151 (1.13)	0.379	50 (1.59)	0.071	591 (1.22)

^a^ represents comparison with No abortion history group

Logistic regression analysis was used to evaluate the association between abortion history and premature delivery, GDM, pregnancy hypertension disorder, PIH, preeclampsia, placenta abnormality, placenta previa, placental accreta, and placental adhesion. The results showed that after adjusting for potential confounding factors (including pre-pregnancy BMI, maternal age, mode of delivery, and parity), differences in placenta abnormality (including placenta previa, placenta accreta, and placental adhesion) still existed. The odds ratios (ORs) and 95% confidence interval (CI) of placenta previa, placenta accreta, and placenta adhesion in pregnant women with only SAB history, only IA history, and both abortions history were 1.294(1.174–1.427), 1.272(1.159–1.396), and 1.390(1.188–1.625), 2.688(1.344–5.374), 2.549(1.268–5.125), and 5.041(2.232–11.386), 2.170(1.872–2.515), 2.028(1.738–2.366), and 3.580(2.917–4.395), respectively. We also found that women with only a SAB history had a higher risk of premature delivery and GDM. However, we did not observe the association between only IA history and premature delivery, GDM, and pregnancy hypertension disorder. The details are shown in Table 3.

**Table 3:** Crude and adjusted OR (95% CI) for the associations between classification of abortions and unfavorable outcomes

	History of abortion (*n* = 27,490)	History of abortion			No abortion history (*n* = 48,283)
		SAB only (*n* = 10,992)	Induced abortion only (*n* = 13,360)	Both SAB and induced abortion (*n* = 3,138)	
Placenta abnormality Crude OR (95% CI)	1.668 (1.572–1.770)	1.642 (1.516–1.778)	1.565 (1.451–1.688)	2.214 (1.961–2.498)	1 (Reference)
Adjusted^a^ OR (95% CI)	1.410 (1.325–1.501)	1.397 (1.286–1.517)	1.338 (1.234–1.451)	1.746 (1.537–1.985)	1 (Reference)
Placenta previa Crude OR (95% CI)	1.536 (1.433–1.647)	1.474 (1.341–1.621)	1.531 (1.403–1.671)	1.775 (1.528–2.062)	1 (Reference)
Adjusted^a^ OR (95% CI)	1.296 (1.205–1.394)	1.294 (1.174–1.427)	1.272 (1.159–1.396)	1.390 (1.188–1.625)	1 (Reference)
Placental accreta Crude OR (95% CI)	5.173 (2.983–8.970)	4.397 (2.244–8.615)	4.469 (2.357–8.473)	10.896 (5.200–22.835)	1 (Reference)
Adjusted^a^ OR (95% CI)	3.014 (1.701–5.338)	2.688 (1.344–5.374)	2.549 (1.268–5.125)	5.041 (2.232–11.386)	1 (Reference)
Placental adhesion Crude OR (95% CI)	2.759 (2.462–3.092)	2.846 (2.467–3.282)	2.261 (1.959–2.610)	4.623 (3.827–5.586)	1 (Reference)
Adjusted^a^ OR (95% CI)	2.272 (2.018–2.559)	2.170 (1.872–2.515)	2.028 (1.738–2.366)	3.580 (2.917–4.395)	1 (Reference)
Premature birth Crude OR (95% CI)	2.205 (1.843–2.638)	1.706 (1.325–2.196)	2.328 (1.880–2.883)	3.440 (2.511–4.712)	1 (Reference)
Adjusted^a^ OR (95% CI)	1.119 (0.930–1.345)	1.384 (1.069–1.792)	0.925 (0.743–1.152)	1.331 (0.962–1.842)	1 (Reference)
Gestational diabetes mellitus Crude OR (95% CI)	1.350 (1.290–1.413)	1.540 (1.450–1.635)	1.162 (1.094–1.234)	1.517 (1.369–1.682)	1 (Reference)
Adjusted^a^ OR (95% CI)	1.141 (1.088–1.198)	1.246 (1.170–1.326)	1.032 (0.967–1.101)	1.125 (1.009–1.255)	1 (Reference)
Pregnancy hypertension disorder Crude OR (95% CI)	1.007 (0.938–1.081)	1.215 (1.107–1.333)	0.841 (0.762–0.928)	0.999 (0.839–1.189)	1 (Reference)
Adjusted^a^ OR (95% CI)	0.983 (0.912–1.059)	1.020 (0.925–1.124)	0.957 (0.863–1.062)	0.964 (0.804–1.156)	1 (Reference)
PIH Crude OR (95% CI)	1.095 (0.990–1.211)	1.355 (1.190–1.542)	0.878 (0.762–1.011)	1.119 (0.880–1.423)	1 (Reference)
Adjusted^a^ OR (95% CI)	1.075 (0.968–1.194)	1.182 (1.034–1.350)	0.971 (0.838–1.125)	1.104 (0.861–1.416)	1 (Reference)
Preeclampsia Crude OR (95% CI)	0.931 (0.843–1.027)	1.083 (0.950–1.235)	0.814 (0.712–0.932)	0.895 (0.699–1.146)	1 (Reference)
Adjusted^a^ OR (95% CI)	0.907 (0.818–1.005)	0.889 (0.776–1.017)	0.948 (0.823–1.092)	0.854 (0.662–1.103)	1 (Reference)

^a^ Adjusted for maternal age, pre-pregnancy BMI, parity (nulliparous, multiparous), and mode of delivery (vaginal delivery, caesarean section)

In addition, considering the strong correlation between abortion history and placenta abnormality, we also conducted a stratified analysis of maternal age and pre-pregnancy BMI to further observe whether the risk of placenta previa, placenta accreta, and placental adhesion changed with maternal age or pre-pregnancy BMI, and we found that compared with women under 35 years old, women over 35 years old with a history of abortion (including SAB and IA) have an increased risk of placenta abnormality, placenta previa and placental adhesion. But we did not find that the association between them changed with pre-pregnancy BMI. See Tables 4 and 5 for details.

**Table 4:** Crude and adjusted OR (95% CI) for the associations between the classification of abortions and unfavorable perinatal outcomes by pre-pregnancy BMI

	History of abortion (*n* = 27,490)	History of abortion			No abortion history (*n* = 48,283)
		SAB only (*n* = 10,992)	Induced abortion only (*n* = 13,360)	Both SAB and induced abortion (*n* = 3,138)	
BMI < 18.5 kg/m2					
Placenta abnormality n (%)	251 (0.91)	79 (0.72)	136 (1.02)	36 (1.15)	366 (0.76)
Crude OR (95% CI)	1.519 (1.287–1.793)	1.316 (1.024–1.691)	1.508 (1.230–1.848)	2.403 (1.675–3.448)	1 (Reference)
Adjusted^a^ OR (95% CI)	1.260 (1.059–1.498)	1.136 (0.879–1.469)	1.259 (1.016–1.561)	1.808 (1.237–2.642)	1 (Reference)
Placenta previa n (%)	189 (0.69)	60 (0.55)	105 (0.79)	24 (0.76)	287 (0.59)
Crude OR (95% CI)	1.448 (1.199–1.747)	1.268 (0.954–1.686)	1.475 (1.173–1.855)	1.986 (1.290–3.057)	1 (Reference)
Adjusted^a^ OR (95% CI)	1.180 (0.970–1.436)	1.103 (0.825–1.475)	1.206 (0.948–1.535)	1.499 (0.957–2.349)	1 (Reference)
Placental accreta n (%)	2 (0.00)	1 (0.00)	1 (0.00)	0 (0)	3 (0.01)
Crude OR (95% CI)	1.442 (0.241–8.632)	2.003 (0.208–19.272)	1.321 (0.137–12.707)	0.000	1 (Reference)
Adjusted^a^ OR (95% CI)	0.843 (0.130–5.475)	1.193 (0.118–12.071)	0.931 (0.087–9.981)	0.000	1 (Reference)
Placental adhesion n (%)	69 (0.25)	17 (0.15)	33 (0.25)	19 (0.61)	59 (0.12)
Crude OR (95% CI)	2.559 (1.803–3.630)	1.741 (1.012–2.995)	2.237 (1.457–3.436)	7.757 (4.571–13.165)	1 (Reference)
Adjusted^a^ OR (95% CI)	2.141 (1.489–3.080)	1.420 (0.815–2.475)	1.866 (1.187–2.932)	5.324 (2.993–9.470)	1 (Reference)
18.5 kg/m2 ≤ BMI < 25 kg/m2					
Placenta abnormality n (%)	1,772 (6.45)	703 (6.40)	806 (6.03)	263 (8.38)	1,876 (3.89)
Crude OR (95% CI)	1.703 (1.592–1.822)	1.679 (1.534–1.837)	1.596 (1.465–1.739)	2.253 (1.966–2.581)	1 (Reference)
Adjusted^a^ OR (95% CI)	1.434 (1.336–1.540)	1.420 (1.294–1.559)	1.362 (1.242–1.492)	1.782 (1.543–2.058)	1 (Reference)
Placenta previa n (%)	1,241 (4.51)	483 (4.39)	595 (4.45)	163 (5.19)	1,389 (2.88)
Crude OR (95% CI)	1.590 (1.470–1.719)	1.536 (1.381–1.708)	1.578 (1.430–1.742)	1.827 (1.545–2.161)	1 (Reference)
Adjusted^a^ OR (95% CI)	1.336 (1.230–1.450)	1.336 (1.198–1.490)	1.306 (1.176–1.451)	1.424 (1.195–1.697)	1 (Reference)
Placental accreta n (%)	47 (0.17)	16 (0.15)	19 (0.14)	12 (0.38)	12 (0.02)
Crude OR (95% CI)	6.829 (3.622–12.876)	5.781 (2.734–12.225)	5.717 (2.774–11.781)	15.168 (6.807–33.797)	1 (Reference)
Adjusted^a^ OR (95% CI)	4.052 (2.105–7.801)	3.518 (1.631–7.591)	3.278 (1.492–7.199)	7.300 (3.013–17.686)	1 (Reference)
Placental adhesion n (%)	592 (2.15)	247 (2.25)	237 (1.77)	108 (3.44)	384 (0.80)
Crude OR (95% CI)	2.732 (2.400–3.110)	2.838 (2.415–3.336)	2.254 (1.914–2.655)	4.397 (3.537–5.466)	1 (Reference)
Adjusted^a^ OR (95% CI)	2.240 (1.956–2.565)	2.163 (1.829–2.558)	2.033 (1.705–2.424)	3.460 (2.734–4.379)	1 (Reference)
BMI ≥ 25 kg/m2					
Placenta abnormality n (%)	220 (0.80)	102 (0.93)	86 (0.64)	32 (1.02)	200 (0.41)
Crude OR (95% CI)	1.535 (1.259–1.871)	1.647 (1.286–2.110)	1.370 (1.055–1.779)	1.720 (1.165–2.539)	1 (Reference)
Adjusted^a^ OR (95% CI)	1.419 (1.155–1.743)	1.481 (1.149–1.910)	1.297 (0.979–1.717)	1.455 (0.966–2.191)	1 (Reference)
Placenta previa n (%)	122 (0.44)	54 (0.49)	52 (0.39)	16 (0.51)	134 (0.28)
Crude OR (95% CI)	1.246 (0.970–1.601)	1.271 (0.920–1.755)	1.222 (0.881–1.694)	1.248 (0.735–2.119)	1 (Reference)
Adjusted^a^ OR (95% CI)	1.121 (0.865–1.453)	1.153 (0.830–1.602)	1.111 (0.783–1.575)	1.023 (0.590–1.774)	1 (Reference)
Placental accreta n (%)	1 (0.00)	0 (0)	1 (0.01)	0 (0)	2 (0.00)
Crude OR (95% CI)	0.679 (0.061–7.487)	0.000	1.562 (0.142–17.245)	0.000	1 (Reference)
Adjusted^a^ OR (95% CI)	0.293 (0.024–3.620)	0.000	0.626 (0.047–8.337)	0.000	1 (Reference)
Placental adhesion n (%)	106 (0.39)	52 (0.47)	37 (0.28)	17 (0.54)	54 (0.11)
Crude OR (95% CI)	2.729 (1.959–3.801)	3.097 (2.104–4.558)	2.176 (1.425–3.323)	3.370 (1.933–5.876)	1 (Reference)
Adjusted^a^ OR (95% CI)	2.582 (1.834–3.635)	2.737 (1.841–4.067)	2.126 (1.346–3.357)	3.046 (1.678–5.530)	1 (Reference)

^a^ Adjusted for maternal age, parity (nulliparous, multiparous), and mode of delivery (vaginal delivery, caesarean section)

**Table 5:** Crude and adjusted OR (95% CI) for the associations between the classification of abortions and unfavorable perinatal outcomes by maternal age

	History of abortion (*n* = 27,490)	History of abortion			No abortion history (*n* = 48,283)
		SAB only (*n* = 10,992)	Induced abortion only (*n* = 13,360)	Both SAB and induced abortion (*n* = 3,138)	
< 35y					
Placenta abnormality n (%)	1,515 (5.51)	619 (5.63)	694 (5.19)	202 (6.44)	2,081 (4.31)
Crude OR (95% CI)	1.539 (1.437–1.648)	1.518 (1.384–1.666)	1.446 (1.324–1.581)	2.087 (1.794–2.428)	1 (Reference)
Adjusted^a^ OR (95% CI)	1.401 (1.306–1.503)	1.399 (1.273–1.537)	1.316 (1.200–1.444)	1.839 (1.574–2.150)	1 (Reference)
Placenta previa n (%)	1,052 (3.83)	421 (3.83)	508 (3.80)	123 (3.92)	1,548 (3.21)
Crude OR (95% CI)	1.421 (1.312–1.540)	1.372 (1.229–1.532)	1.414 (1.276–1.567)	1.662 (1.376–2.007)	1 (Reference)
Adjusted^a^ OR (95% CI)	1.288 (1.187–1.399)	1.290 (1.154–1.442)	1.254 (1.127–1.395)	1.448 (1.193–1.756)	1 (Reference)
Placental accreta n (%)	25 (0.09)	10 (0.09)	8 (0.06)	7 (0.22)	9 (0.02)
Crude OR (95% CI)	5.730 (2.674–12.278)	5.539 (2.250–13.634)	3.776 (1.457–9.790)	15.942 (5.931–42.848)	1 (Reference)
Adjusted^a^ OR (95% CI)	4.334 (1.991–9.435)	4.654 (1.881–11.517)	2.626 (0.959–7.187)	12.855 (4.577–36.102)	1 (Reference)
Placental adhesion n (%)	499 (1.82)	208 (1.89)	201 (1.50)	90 (2.87)	409 (0.85)
Crude OR (95% CI)	2.551 (2.236–2.910)	2.570 (2.171–3.042)	2.108 (1.778–2.500)	4.650 (3.686–5.868)	1 (Reference)
Adjusted^a^ OR (95% CI)	2.302 (2.011–2.634)	2.227 (1.878–2.641)	2.019 (1.689–2.412)	4.055 (3.182–5.168)	1 (Reference)
≥ 35y					
Placenta abnormality n (%)	728 (2.65)	265 (2.41)	334 (2.50)	129 (4.11)	361 (0.75)
Crude OR (95% CI)	1.551 (1.359–1.770)	1.605 (1.357–1.897)	1.446 (1.237–1.691)	1.759 (1.420–2.178)	1 (Reference)
Adjusted^a^ OR (95% CI)	1.535 (1.341–1.756)	1.524 (1.286–1.805)	1.412 (1.196–1.667)	1.698 (1.364–2.114)	1 (Reference)
Placenta previa n (%)	500 (1.82)	176 (1.60)	244 (1.83)	80 (2.55)	262 (0.54)
Crude OR (95% CI)	1.442 (1.236–1.683)	1.437 (1.179–1.752)	1.442 (1.203–1.727)	1.455 (1.122–1.888)	1 (Reference)
Adjusted^a^ OR (95% CI)	1.402 (1.199–1.640)	1.414 (1.158–1.727)	1.336 (1.104–1.616)	1.380 (1.059–1.798)	1 (Reference)
Placental accreta n (%)	25 (0.09)	7 (0.06)	13 (0.10)	5 (0.16)	8 (0.02)
Crude OR (95% CI)	2.309 (1.041–5.125)	1.829 (0.663–5.051)	2.461 (1.019–5.944)	2.914 (0.915–8.926)	1 (Reference)
Adjusted^a^ OR (95% CI)	2.237 (0.999–5.007)	1.593 (0.574–4.421)	2.732 (1.062–7.030)	2.571 (0.819–8.066)	1 (Reference)
Placental adhesion n (%)	268 (0.97)	108 (0.98)	106 (0.79)	54 (1.72)	88 (0.18)
Crude OR (95% CI)	2.301 (1.803–2.936)	2.641 (1.984–3.517)	1.849 (1.388–2.462)	2.957 (2.092–4.179)	1 (Reference)
Adjusted^a^ OR (95% CI)	2.365 (1.845–3.032)	2.352 (1.761–3.143)	2.047 (1.503–2.788)	3.104 (2.168–4.443)	1 (Reference)

^a^ Adjusted for pre-pregnancy BMI, parity (nulliparous, multiparous), and mode of delivery (vaginal delivery, caesarean section)

## Discussion

As far as we know, this was the first large-scale study in China to explore the association between the history of abortion and perinatal outcomes after singleton pregnancies. After analyzing 75,773 women with singleton pregnancies, we found that women with only SAB history before pregnancy had a higher risk of premature delivery, GDM, placenta abnormality, placenta previa, placenta accreta, and placental adhesion in subsequent pregnancies. Women who only had IA history and had both SAB and IA history were at greater risk of placenta abnormality, placenta previa, placenta accreta, and placenta adhesion in subsequent pregnancies.

With regard to SAB, recurrent abortion was the most studied. Many studies have shown that recurrent abortion was associated with a variety of adverse obstetrical outcomes, including preeclampsia, premature delivery, small for gestational-age infants, placental abruption, pregnancy complications related to placental dysfunction, etc. [14–16]. A new study in China has found that women with a history of SAB had an increased risk of GDM in subsequent pregnancies [17]. An Iranian study assessed the history of SAB and the risk of preterm birth in subsequent pregnancies. The results showed that the history of SAB was related to the increased probability of preterm delivery, that is, the more times of SAB, the greater the probability of preterm delivery [18], and other studies had similar results [19]. These findings were consistent with our conclusions because we found that women who had only SAB history before pregnancy had a higher risk of preterm delivery and GDM in subsequent pregnancies after adjusting for confounding factors. Although we did not observe that the history of IA was related to premature delivery and GDM, other studies suggested that the risk of premature delivery increased after IA [20–22]. It has been reported that women with abnormal glucose metabolism before pregnancy will have an increased risk of miscarriage in the following pregnancy [23, 24], which may explain the increased risk of GDM after SAB. However, because this study is retrospective and lacks enough detailed glucose metabolism information, we are not sure whether it is related to this reason. We speculate that the reason for the increase in the incidence of preterm delivery may be that the history of abortion could lead to cervical insufficiency and increase the risk of infection [25, 26]. However, there is no literature report on why the risk of premature delivery of SAB increases, but the risk of IA does not increase significantly.

We found that pregnant women with only SAB history were more likely to have PIH and preeclampsia, but pregnant women with only IA history were less likely to have PIH and preeclampsia, and these results were statistically different. After Logistic regression analysis, these differences still existed and had statistical differences. However after adjusting the potential confounding factors, these differences disappeared. Beck et al. found that the probability of preeclampsia in pregnant women with IA was significantly lower than that in pregnant women without abortion or with SAB history [27], and other studies had similar results [28, 29]. These were all consistent with our research results. However, other studies have suggested that a history of SAB can reduce the risk of preeclampsia [30, 31]. It has been suggested that normal pregnancy interrupted in the early stage of pregnancy may cause immune changes, thereby reducing the risk of preeclampsia in subsequent pregnancy [29]. Other studies have suggested that the interval between two pregnancies may be a major determinant [32, 33]. However, the mechanism of the relationship between SAB, IA and pregnancy induced hypertension needs to be further explored.

With regard to placenta abnormality, we mainly explored four placenta-related complications: placenta previa, placental abruption, placental accreta, and placental adhesion. We found that whether there was only a history of SAB, only a history of IA, or a history of both SAB and IA, they were associated with an increased risk of placenta previa, placental accreta, and placental adhesion, but not with placental abruption. Our conclusions were consistent with those of other studies [34]. Studies by W Zhou et al. have shown that there was a positive correlation between abortion and placental residues in subsequent singleton live births [35]. Zhu et al.'s study showed that women with a history of IA had a higher incidence of placental abruption. However, after considering confounding factors, the difference was not statistically significant [36]. In addition, other studies also believed that placenta previa, placenta residue, and other placenta abnormality were related to abortion [37, 38]. The placenta abnormality occurred in the subsequent pregnancy after abortion, and the mechanism may be that the operation damaged the endometrium and uterine cavity [39, 40].

Our study was the first large-scale study in China to explore the association between the history of abortion and multiple pregnancy complications after singleton pregnancy. However, our research had some limitations. First of all, this was a retrospective study. Abortion information was self-reported by pregnant women, and there may be underreporting. Secondly, some literature believed that the pregnancy interval after abortion will have an impact on perinatal complications [41], but we did not study the effect of pregnancy interval after abortion on the outcomes. Thirdly, we did not classify induced abortion to study the effects of drug abortion and surgical abortion on the results [42]. Finally, because the electronic medical record system did not record the gestational age at the time of abortion in detail and lacked the history of PE, GDM and placenta abnormality in their previous pregnancy, we did not study its influence on the pregnancy outcomes.

## Conclusion

In a word, our research showed that pregnant women who had a history of abortion before pregnancy (including a history of SAB or IA) and then got singleton pregnancies were more likely to have premature delivery, GDM, placenta abnormality, placenta previa, placenta accreta, and placenta adhesion. After adjusting for confounding factors, it was found that the history of SAB and IA were still associated with placenta abnormality, placenta previa, placental accreta, and placental adhesion.

## Data Availability

The datasets used and analyzed during the current study are available from the corresponding author upon reasonable request.
